# Narrative Constructions for the Organization of Self Experience: Proof of Concept via Embodied Robotics

**DOI:** 10.3389/fpsyg.2017.01331

**Published:** 2017-08-15

**Authors:** Anne-Laure Mealier, Gregoire Pointeau, Solène Mirliaz, Kenji Ogawa, Mark Finlayson, Peter F. Dominey

**Affiliations:** ^1^Human and Robot Cognitive Systems, Stem Cell and Brain Research Institute U1208, Institut National de la Santé et de la Recherche Médicale Lyon, France; ^2^Computer Science Department, Ecole Normale Supérieure de Rennes Rennes, France; ^3^Graduate School of Letters, Hokkaido University Sapporo, Japan; ^4^School of Computing and Information Sciences, Florida International University Miami, FL, United States

**Keywords:** narrative, grammatical construction, function word, reservoir computing, situation model, human-robot interaction, narrative enrichment

## Abstract

It has been proposed that starting from meaning that the child derives directly from shared experience with others, adult narrative enriches this meaning and its structure, providing causal links between unseen intentional states and actions. This would require a means for representing meaning from experience—a situation model—and a mechanism that allows information to be extracted from sentences and mapped onto the situation model that has been derived from experience, thus enriching that representation. We present a hypothesis and theory concerning how the language processing infrastructure for grammatical constructions can naturally be extended to narrative constructions to provide a mechanism for using language to enrich meaning derived from physical experience. Toward this aim, the grammatical construction models are augmented with additional structures for representing relations between events across sentences. Simulation results demonstrate proof of concept for how the narrative construction model supports multiple successive levels of meaning creation which allows the system to learn about the intentionality of mental states, and argument substitution which allows extensions to metaphorical language and analogical problem solving. Cross-linguistic validity of the system is demonstrated in Japanese. The narrative construction model is then integrated into the cognitive system of a humanoid robot that provides the memory systems and world-interaction required for representing meaning in a situation model. In this context proof of concept is demonstrated for how the system enriches meaning in the situation model that has been directly derived from experience. In terms of links to empirical data, the model predicts strong usage based effects: that is, that the narrative constructions used by children will be highly correlated with those that they experience. It also relies on the notion of narrative or discourse function words. Both of these are validated in the experimental literature.

## Introduction

This research is situated in the developmental context of the narrative construction of reality as proposed and developed by Bruner et al. (Bruner, 1990, 1991, 2009; Fivush, 1991, 1994; Neisser, 1997; Nelson and Fivush, 2004). The idea is that as the child experiences interaction with people and objects in the world, adults provide narrative structure that allows the child to organize its internal representation of this experience in a more meaningful way. This includes the understanding of other's behavior in terms of possibly unseen intentional states. In order for this representation of causal relations between intentional states and actions to be extracted from language, some mechanism for performing this representation building must exist. Here we develop a hypothesis and a corresponding model, of a mechanism for narrative construction processing that performs this function. The key notion here is that narrative provides a structure that enriches experience.

What then is narrative? Narrative is possibly one of the most complex of human mental and cultural achievements, perhaps because it is the substrate that we use for communicating what is most important to us. Against a background of the ordinary and canonical events, narrative is interpreted to give meaning to breaches in and deviations from “normal” states of affairs. Narrative structure can have rich complexity (McCabe and Peterson, 1991). Efforts have been made to formally characterize narrative structure, for example in terms of story grammars (Propp, 1968; Mandler and Johnson, 1977), and state of the art AI and machine learning has been used to extract the analogical structure from Russian folktales initially inspired by the work of Propp (Finlayson, 2012). Scholarly volumes have been dedicated to the elaboration and characterization of narrative structure (McCabe and Peterson, 1991).

In the face of this complexity, we refine our question and ask what is narrative in the context of the developing child and the narrative construction of reality? In an analysis of the narrative construction of the self, Menary (2008) notes Lamarque's minimal definition of narrative where “at least two events must be depicted in a narrative and there must be some more or less loose, albeit non-logical relation between the events. Crucially, there is a temporal dimension in narrative” (Lamarque, 2004, p. 394). In her characterization of how the child begins to go beyond a purely canonical representations of its life events, Nelson states that “Narrative is the vehicle of communicating representations of events between people by verbal means” (Nelson, 2003, p. 32). The word “narrative” will be used in this context, as a form of discourse that communicates experience of a causally or temporally related chain of events in terms of human goals, actions, and intentions. Likewise, narrative structure will refer to the ensemble of structured relations between events, defined in terms of five dimension of time, space, causality, intentionality, and protagonist (Zwaan et al., 1995). A hypothesis about how narrative constructions can be extended from grammatical constructions is developed, and an implemented proof of concept system that allows a humanoid robot to begin to make sense of its experience in this context is presented.

The objective is not to account for all of the rich dimensions of narrative structure. Rather, the current goal is to propose a mechanism that can extract meaning from simple narrative and map it onto meaning representations, enriching these representations, with a particular focus on relations between events and intentional states that might not be visibly observable in behavior. The motivation is that narrative provides a framework for making events more meaningful in terms of human intention and motivation (Bruner, 1990, 1991, 2009). It connects individual events and the corresponding sentences into larger wholes, and is necessary for constructing and expressing complex meanings that include intention, motivation and the intentional role of the self (Bruner, 1991; Nelson and Fivush, 2004). Importantly, this is consistent with the notion that narrative and such thought processes can be distinct (Fedorenko and Varley, 2016), and that narrative can provide crucial input to these systems.

### Usage-based learning in human development

This raises the question of how narrative constructions are acquired by children. To begin to address this, one can exploit the analogy with usage-based learning of grammatical constructions. Usage-based learning is a social-pragmatic approach to language acquisition in which children learn linguistic structures through intention-reading and pattern-finding in their discourse interactions with others (Tomasello, 2003). From the outset of life, children and adults interact in feeding, changing diapers, getting ready for bed, etc. in repeating rituals that are accompanied by language from the adults (Clark, 2003). This provides a rich shared space where language can enrich meaning at multiple levels (Nomikou et al., 2016). In the usage-based concept of language acquisition, the child's first words allow reference to people and objects that are of central prominence in everyday life (Clark, 2003; Tomasello, 2003). After words, the first grammatical constructions are fixed around specific verbs, and specific actions that tend to be repeated and ritualized in the infants' social environment (Tomasello, 2000, 2003; Lieven et al., 2003). Constructions then become more abstract and generalized as the child's world becomes enriched (Goldberg, 1995; Tomasello, 2003). Narrative constructions are a further level in this successively elaborated structure. Whereas, grammatical constructions establish relations between an event and its argument components, narrative constructions establish relations between multiple events. In the usage-based context, this would begin with narrative constructions which will be specific to particular types of repetitive ritualized interactions. This provides the basis for generalization across constructions, as was observed at the sentence level, with grammatical constructions in children (Tomasello, 2000, 2003) and in sentence processing models (Hinaut and Dominey, 2013; Hinaut et al., 2015).

The grammatical style used by caregivers is directly visible in the language production of children (Tomasello, 2000). One would expect the same for narrative constructions. Indeed, such a usage-based approach to narrative construction learning is supported by experimental evidence which indicates that the narrative style used by caregivers to describe past events with their children influence the style that is subsequently adopted by the children (Fivush, 1991, 1994; Nelson and Fivush, 2004). This can be summarized by the observation that “By participating in adult-guided conversations about their past experiences, children are learning the culturally appropriate narrative forms for recounting the past” (Fivush, 1994, p. 137). Likewise, it has also been observed that listening to maternal story-telling has an immediate effect on children's own storytelling. In particular, the frequency of use of evaluative expressions (reference to internal states of actors, of the storyteller, reference to absent actors) in the child narrative is correlated to that in the maternal narrative (Harkins et al., 1994). These data indicate that similar to the usage based development of grammatical construction knowledge, there is a usage based development of narrative construction knowledge.

In addition to this evidence for reuse of narrative constructions, Lever and Sénéchal (2011) demonstrated that children's narrative production style was influenced by their participation with adults in a dialogic interaction during story reading. In this interaction, as the story was being read, the teacher prompted the child to answer questions, followed correct answers with expansions, helping as needed, etc. Children that underwent 8 weeks of dialogic story telling training demonstrated in subsequent narrative production a significant increase in the inclusion of story grammar units related to internal responses, internal plans and reactions, that control children did not.

### Toward modeling

Thus, language shapes and enriches meaning from the outset of learning. In this context we previously demonstrated how a robotic system can learn to map lexical elements onto physical action and predicate representations, and how language can provide a perspective focus that could be considered a first primitive form of enrichment (Dominey and Boucher, 2005; Mealier et al., 2016). Here our focus is on later development, where this enrichment takes place at the level of narrative. Once words and grammatical constructions can be learned, narrative can further enrich meaning. Models of language that are based on interaction with the world, particularly the embodied construction grammar approach (Bergen and Chang, 2005; Lakoff and Narayanan, 2010), allow the grounding of meaning in experience as coded in image or conceptual schemas, but there is still a need for a method for acquiring novel narrative schemas, and ways of organizing these schemas into larger wholes. In this context the current research develops a model of narrative structure learning and provides specific examples of how learned narrative structure can enrich understood and communicated meaning.

Going beyond purely simulation-based validation, the narrative construction (NCx) model is then embedded in a humanoid robot cognitive system. The EU project WYSWYD (What you say is what you did) provides an infrastructure for goal oriented, motivated behavior with others (Lallee and Verschure, 2015). Part of this infrastructure includes development of an autobiographical memory that encodes the extended experience of the iCub during interaction with humans, allowing it to learn actions and cooperative plans (Pointeau et al., 2013, 2014), and forming the basis for imposing narrative structure upon experience.

The approach can be illustrated with a behavioral scenario or situation that can give rise to a simple complication-resolution narrative. Humans represent situations in terms of five dimensions of time, space, causality, intentionality, and protagonist (Zwaan and Radvansky, 1998). These dimensions define links between events that provide the content that is to be expressed in the narrative. In the example scenario a humanoid robot, the iCub, is trying to grasp an object, it fails, and then asks another agent to give him the object, which the second agent does. The iCub now has now achieved his initial goal. Here is the corresponding narrative that could describe such a situation:

*I wanted to get the giraffe. But I failed to grasp it because it was out of reach. So I found a different action. If I could ask you to give it to me, then you would give it to me. So I asked you to give it to me, and you gave it to me. I have the giraffe now because I asked you to give it to me*.

According to our minimalist definition of narrative (Nelson, 2003; Lamarque, 2004; Menary, 2008), that preferentially addresses natural narratives as they occur in everyday conversations (Van Dijk, 1975) this narrative employs the ordered triple of exposition, complication, and resolution. The exposition specifies the desire to have the giraffe. The complication is the failure to grasp the giraffe, and the resolution is the alternative solution of asking for help. This narrative is of interest because it specifies temporal, causal, and intentional links between events in the interactions through the use of the narrative function words *but, so, then, now*, and *because*. The notion of narrative function word merits a clarification. In the same way that grammatical function words like “to” and “by” introduce grammatical relations between words within grammatical constructions, narrative function words like “because” and “then” introduce causal and temporal relations between events in narrative structure. Such temporal and causal relationships are necessary (but not completely sufficient) for creating meaning in narrative structure. Importantly, we do not claim that there are specific words that will create the more elaborate narrative structures such as the “villainy,” or “revenge” themes. Indeed, Finlayson (2012) demonstrated how such themes can be defined in terms of a multilayered and hierarchical morphological structure, including event subtypes such as reward, punishment, struggle, victory, and roles such as hero and villain. Narrative function words specify temporal and causal relations at the service of narrative structure, but they do not directly invoke such event subtypes and roles.

**Figure 1:**
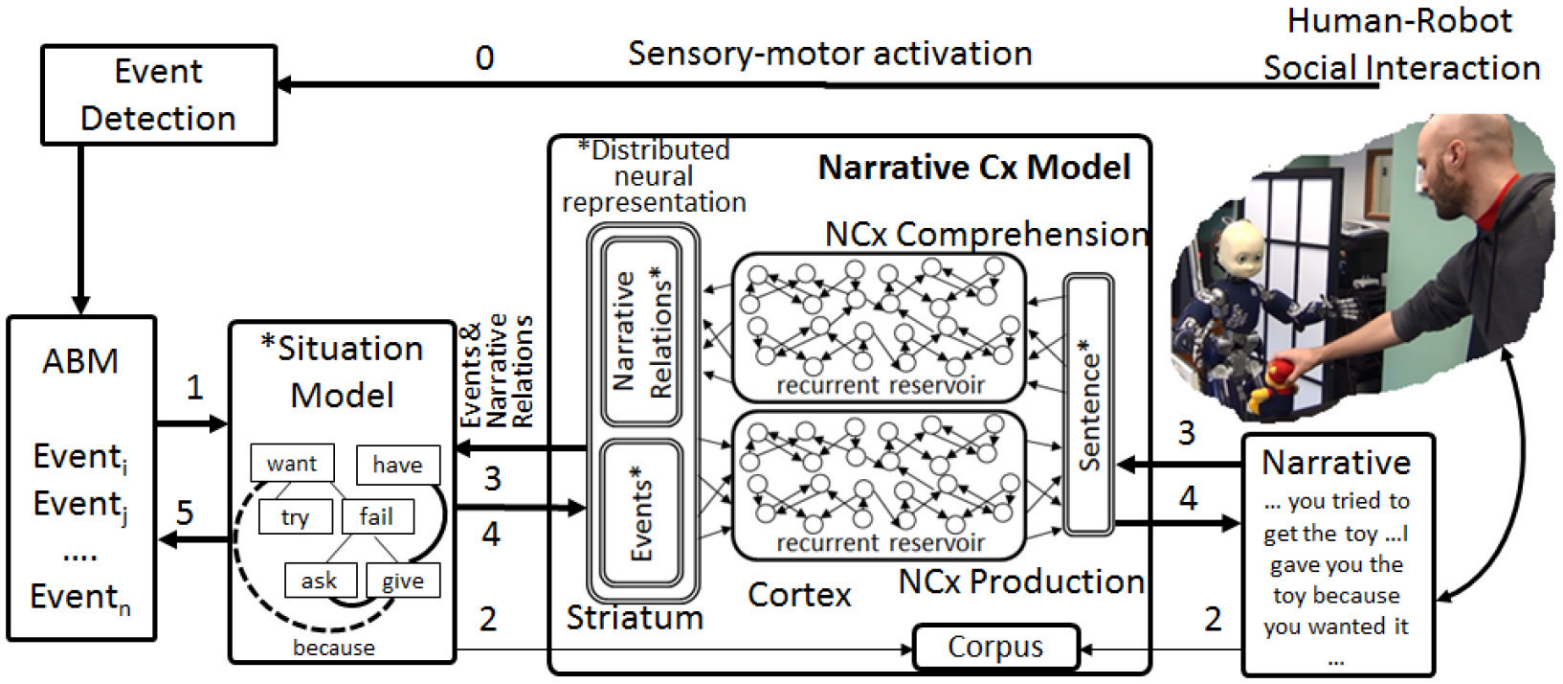
Narrative processing system overview. Implemented Embedding of Narrative Construction (NCx) Model in the iCub cognitive system. Encoding experience in the ABM and situation model. The robot interacts with humans via our extended cooperative interaction infrastructure (Lallée et al., 2012, 2013; Petit et al., 2013; Mealier et al., 2016) (not shown here). 0—Propositional representations extracted by perceptual processing for Event Detection and encoded in the Auto-Biographical Memory (ABM) (Pointeau et al., 2014). 1—Events encoded in the ABM are directly converted into Initial State, Goal, Action, Result, Final state (IGARF) representations in the Situation Model. Thus, the robot's experience is encoded the ABM, and provisionally in the situation model. Enrichment of experience by narrative: The human narrates the interaction. 2—Sentences in the narrative are aligned with meaning coded in the Situation Model in order to generate a corpus of sentence-meaning pairs that is used to train the comprehension and production models. 3.1 The narrative that describes the observed events is processed by the trained comprehension model, and the additional relational links coded in the narrative are used to enrich the Situation Model (e.g., dotted “because”). 3.2 Based on a new narrative that matches a learned narrative construction, the system can create a new situation model (inheriting narrative relations). 4. Once a situation model has been instantiated, the system extracts the meaning to the narrative production model to generate the corresponding narrative. 5. Once a situation model has been enhanced by narrative, its contents can be written into the ABM as an enriched experience—thus narrative enriches and modifies experience (not implemented). New experiences that overlap with learned situation models will inherit the enriched narrative structure (^*^indicates reservoir-compatible distributed neural code).

In order to characterize how narrative enriches meaning, we start with an overview of how narrative production and comprehension fit into an ecological setting where a cognitive system interacts with its environment in a simplified and selective modeling context illustrated in Figure 1. The operation of the system is based on concepts from human development, where parent/caretaker interactions provide specific structuring of events into narrative to describe and enrich experience shared by the parent and child. There is a strong relation between the structure of this child-directed narrative, and the subsequent use of narrative by the child (Fivush, 1991, 1994; Nelson and Fivush, 2004). The modeling exercise is based on the following assumptions:

1. The child has access to the meaning of certain canonical events (Bruner, 1990, 1991) via the perceptual system in the form of image schemas (Mandler, 2012).
2. The parent provides a structured narrative of these events that can enrich the child's representation of meaning (Nelson and Fivush, 2004).
3. This input provides the basis of a structured learning corpus.
4. Relations that are implicit in the perceived events are made explicit by their association with narrative functions words.
5. Such assumptions should apply across languages, and should not rely on specificities of English or any particular language.

It is important to note that while we present these assumptions as distinct elements that will contribute to the development of our computational model, we fully subscribe to the notion that in human development these steps overlap and interact from the outset. These assumptions represent a schematization and simplification of the developmental processes that allow a functional implementation in the model cognitive system. We now provide a walkthrough of how this works in the model, illustrated by Figure 1, with a detailed description of the implementation of the system and its operation in Section From Grammatical Construction to Narrative Construction.

**Figure 2:**
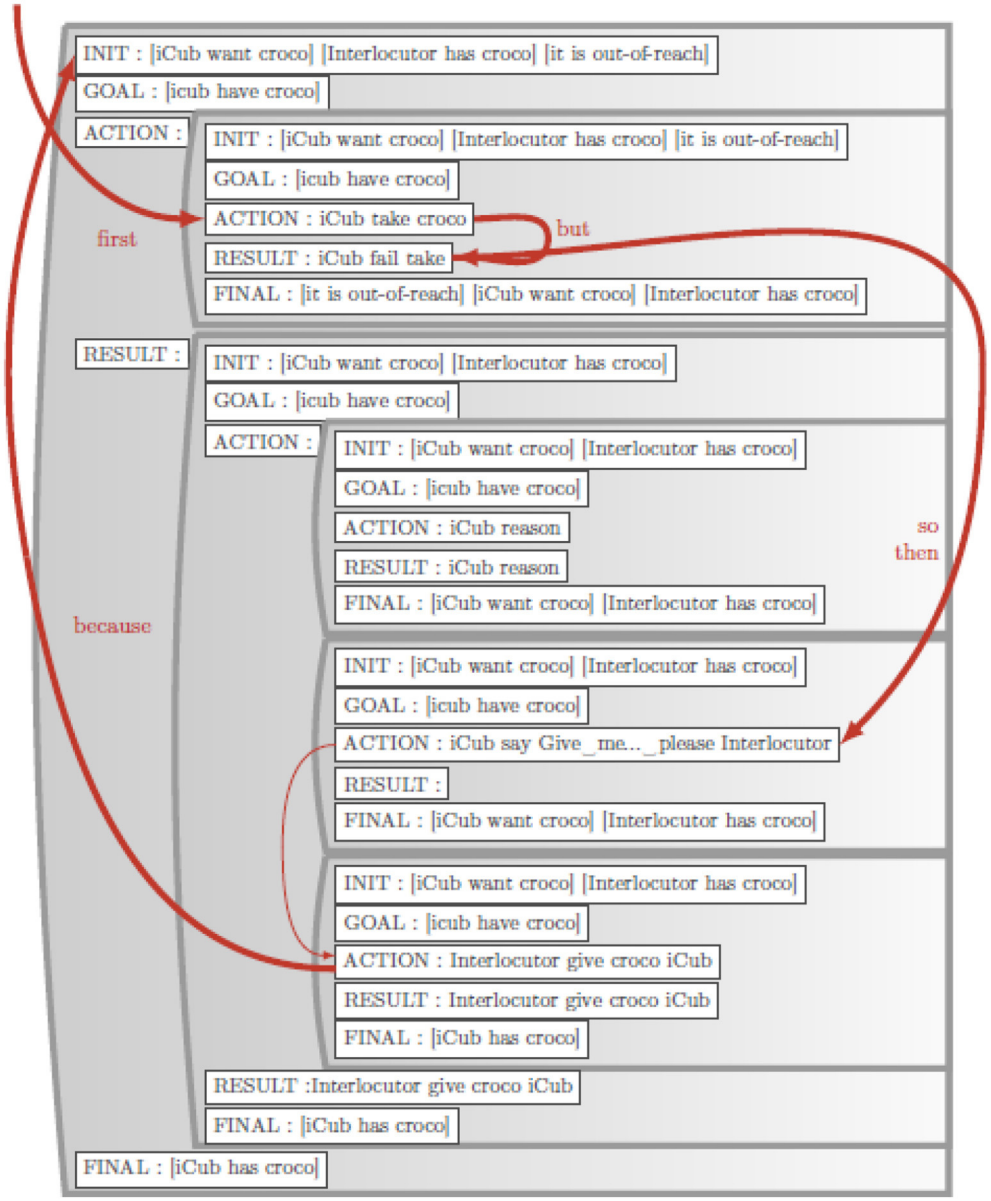
Situation model contents example. Example of a situation model created automatically from the ABM via the ABMtoSM module. The narrative links (in red) were created during the Situation Model Enrichment via narrative with the NCx to SM module.

The robot enacts the scenario described above, first failing to grasp the toy, and finally getting help from the human. This populates the autobiographical memory (ABM) with event representations which encode all of the robot's experience, including interaction with the human. This experience is transformed into a situation model representation in which events are coded in terms of initial state, goals, action, result and final state. The human then provides a narrative that enriches the experience of what happened. The sentences in the narrative are matched to their corresponding events in the situation model to generate a paired sentence-meaning corpus that can then be used to train the comprehension and production models. Narrative function words are detected and used to create narrative links, crucially imposing additional meaning that was not there in the original experience. For example, “John gave Mary the ball because she wanted it” expresses a causal relation that is not necessarily visible in the physical events. Once the comprehension model is trained, the same narrative can then be used to enrich the meaning that was initially formed purely from observed experience, by extracting these additional relations. In this case, such an enrichment is seen with the narrative function word “because.” Likewise, the system can now use the trained production model to generate narrative from the Situation Model in a process similar to construal (Mealier et al., 2016). A situation model corresponding to this narrative is illustrated in Figure 2. Narrative links added by narrative enrichment are shown in red. Given this overview, more detail on the functional elements in Figure 1 are now provided. The initial focus is on the operation of the narrative construction (NCx) comprehension and production models, starting with an introduction to the grammatical construction (GCx) models from which they will derive.

## A neurocomputational model of grammatical constructions

This implementation of narrative constructions builds on the clearly established notion of grammatical construction (Goldberg, 1995, 2003) that implements the relatively direct mapping between sentence structure, and the argument structure of events. This corresponds closely to the notion of expressing and determining *who did what to whom*. We first developed a recurrent network model for learning grammatical constructions (Dominey, 2001, 2003, 2013; Dominey et al., 2003) as the mapping from sentence form to a predicate-argument representation of meaning in the context of physical events and action (Dominey and Boucher, 2005). We have subsequently improved and studied generalization in such models for sentence comprehension and production (Hinaut and Dominey, 2013; Hinaut et al., 2015). These models operate on the principal that thematic roles in sentences are specified by multiple cues including word order and grammatical function words (e.g., *was, to, by, from, that*, etc.), in the context of Bates' and MacWhinney's cue competition hypothesis (Bates et al., 1982, 1991; Bates and MacWhinney, 1987; Li and MacWhinney, 2013). One of the objectives of the cue-competition model was to account for how these different cues would arise in different configurations across languages. We have demonstrated how closed class structure allows the assignment of thematic roles in English and Japanese for sentence comprehension (Hinaut and Dominey, 2013) and production (Hinaut et al., 2015), consistent with the cue competition hypothesis. Here we will extend this to narrative construction processing for comprehension and production.

### Sentence comprehension

**Figure 3:**
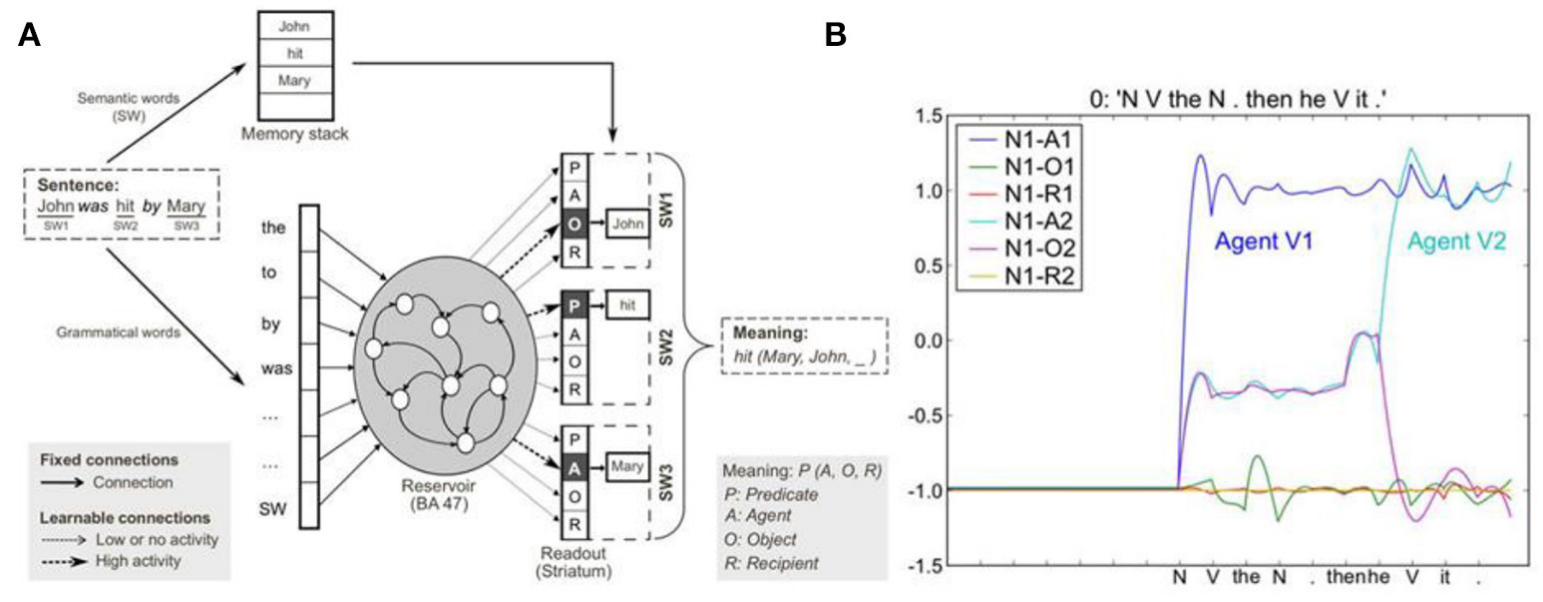
Sentence comprehension model. **(A)** Grammatical construction processing in the reservoir framework. Semantic and grammatical words (i.e., open and closed class words, respectively) are separated on input. Semantic words (SW) are stored in a memory stack. Grammatical words and a single input for all SWs are inputs to the reservoir (analogous to prefrontal cortex area BA47). During training, input sentences are presented word-by-word, and readout units (corresponding to striatum) are forced to the corresponding coded meaning (i.e., SW1-Object, SW2-Predicate, SW3-Agent). In testing, readout units code the predicted role(s) of each semantic word, forming the coded meaning. The meaning [i.e., hit(Mary, John, _)] can be reconstructed from the coded meaning, as SWs in memory stack are reassigned to the thematic roles (predicate, agent, object, recipient) identified in the read-outs. **(B)** Activation of readout neurons coding the semantic role of the first noun (N1) in the mini-discourse “John threw the boomerang. Then he caught it.” The readout neurons indicate that the first noun, John, is the agent of action 1, and the agent of action 2 (from Hinaut and Dominey, 2013).

**Figure 4:**
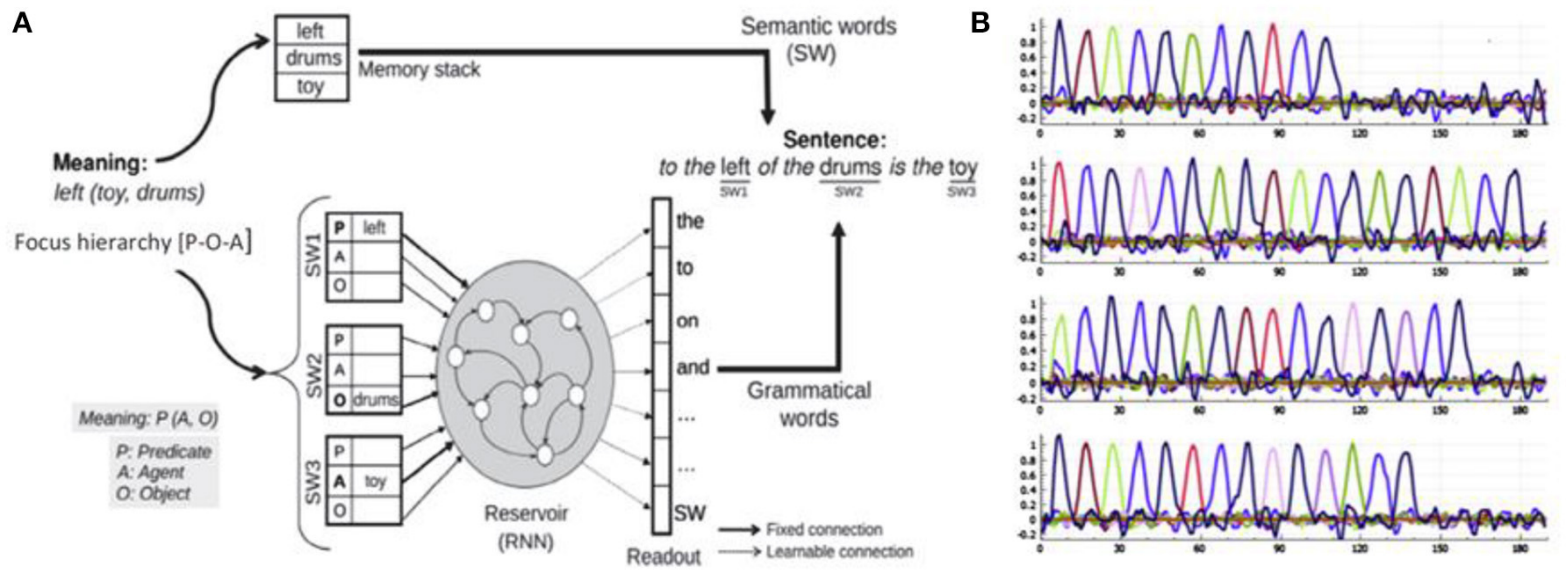
Sentence production model. **(A)** The input should express two aspects of the meaning: (1) meaning format [Predicate(Agent, Object)—left(toy, drums)] corresponding to relation toy to the left of drums, and (2) the focus hierarchy indicating [SW1—Predicate, SW2—Object, SW3—Agent] which could be written in a compact way a [P-O-A]. The system must find a construction that allows this mapping of SWs to thematic roles. **(B)** Sample activation of readout neurons for 4 different sentences.

Figures 3, 4 illustrate the original grammatical construction comprehension and production models. The comprehension model learns to extract thematic roles including predicate, agent, object, and recipient, from an unrestricted variety of different sentence forms. Learning is based on training corpora made of sentences paired with meanings specified in terms of predicate, agent, object, recipient event structures. Grammatical constructions are thus mappings between sentence form and meaning. The essential idea is that the sentence form corresponds to the pattern of semantic words (open class words) and grammatical function words (closed class words) that are provided to a recurrent neural network as input, in the order they arrive, by activation of corresponding input neurons. The recurrent network is built from leaky integrator neurons that have fixed time constants that govern the rapidity of their activation. These neurons are interconnected by fixed excitatory and inhibitory connections, providing the recurrent reservoir with a rich dynamics that is sensitive to the order and temporal structure of the input sequences (Dominey, 1995; Dominey et al., 1995). The model is trained to associate these rich states of activation, driven by the sentence form, with the activation of output (readout) neurons that code the thematic roles of the semantic words in the sentence. In the example sentence “John was hit by Mary” semantic word 1 (SW1) is the object (O), SW2 the predicate (P), and SW3 the agent (A). Thus the recurrent reservoir model learns to associate sentence forms with semantic role labeling. Importantly, the model can generalize to new constructions that were not present in the training set (Hinaut and Dominey, 2013).

### Sentence production and the focus hierarchy

Figure 4 illustrates how this same framework has been reversed for sentence production. Interestingly, we identified a form of asymmetry between production and comprehension. For comprehension, the input is a sentence, and the output is the activation of neurons coding the semantic roles of the different open class words. For production one would think that specification of the intended meaning purely in terms of who did what to whom would be sufficient to allow generation of the sentence, but this is not quite enough. A given meaning can be specified in multiple ways (e.g., the active and passive sentence forms), so additional information is required to constrain the desired sentence. That is, in language production, the system must accommodate the multiple possible construals of a mental model, and be capable of generating the corresponding sentences for expressing them. This additional information is referred to as the focus hierarchy (Hinaut et al., 2015). Construal and the focus hierarchy are first considered in the broader context of phrasal semantics, the meaning pole in the form-meaning characterization of grammatical constructions. In Jackendoff's characterization (Jackendoff, 2002) phrasal semantics can be considered to include three levels: The descriptive tier addresses the organization of conceptual functions, arguments, and modifiers, including thematic role assignment. The referential tier identifies how constituents refer to conceptualized individuals in an ordered, indexed manner, with lists of indices that record the active referents in the discourse. This is related to the notion of the indexed or ordered set of open class items in the focus hierarchy, and would include pronoun reference. The information structure tier characterizes distinctions including focus/presupposition, theme/rheme, and old/new information, in the context of how the speaker intends to inform the listener in the context of the previous discourse. The focus hierarchy is part of the information structure tier, and it also contains information in the descriptive tier, related to thematic role assignment. It specifies the semantic roles (predicate, agent, object, recipient, or PAOR) of the semantic words, in the order that they appear in the sentence. As illustrated in Figure 4, the construal that is provided as input to the production model is thus the situation model or mental model combined with a focus hierarchy (Hinaut et al., 2015). This allows the production model to make a well-formed selection of the construction to use for expressing a given construal.

### Argument generalization

Clearly one of the most productive and interesting aspects of language is its generative nature, which takes place in at least two distinct ways. The first we will refer to as *argument generalization*. This means that grammatical constructions that have been learned with a specific set of arguments, e.g., “We're going to eat now” can be extended to take new arguments, e.g., “We're going to play now” and the meaning can be generalized to the new argument (Dominey et al., 2009). This corresponds to the verb island hypothesis where children can begin to form argument slots around specific verbs (Tomasello, 2000), and then liberate this fixed structure for generalized argument constructions (Goldberg, 1995). In a sense one can think of the construction as a computer program or macro, and the open class or semantic words that populate it as arguments to the program. This form of argument generalization becomes potentially more interesting at the level of the narrative construction, since the narrative is a much richer program, composed of a number of interlinked programs (sentences). Thus when a new argument is inserted into an existing narrative structure, the potential new meaning that will be generated can be significant, as will be demonstrated for metaphor and analogy below. The second form of generativity at the sentence level is the ability to understand and to generate sentences for which there is not an existing example in the learned construction inventory (Elman, 1990, 1991; Miikkulainen, 1996; Voegtlin and Dominey, 2005). In the domain of sentence comprehension and production it has been demonstrated that with a corpus that is sufficiently large to capture the variability of the underlying grammar, the models presented in Figures 3, 4 demonstrate robust generalization to understanding sentences whose underlying construction was not provided in the training corpus (Hinaut and Dominey, 2013; Hinaut et al., 2015). The focus in this paper will be on argument generalization. However, it has been demonstrated that by learning how distinct narrative function words are used, the system can generalize and create new narrative constructions (Pointeau et al., in preparation).

The objective of this research is to develop and demonstrate a working neural model that can learn to extract meaning from narrative, and to generate narrative from meaning. The following sections will explain the architecture and function of the narrative construction models. It will then be illustrated how the models learn to understand and then produce narratives. This will lead to demonstration of argument generalization and how this allows the system to generate novel narratives. It will be shown how this work is naturally extended to ever higher levels of language meaning, such as metaphor, and analogy, illustrating additional manners in which constructions are used to make meaning. In order to provide a computational framework in which the model is situated, we will illustrate how these functions have been implemented in a humanoid robot, which allows a context in which the training corpora are naturally generated as part of the human-robot interaction.

## From grammatical construction to narrative construction

The hypothesis that we test in the current research is that this notion of construction as form to meaning mapping can be extended to the notion of narrative construction. A grammatical construction maps a sentence to a meaning, specified as one or more PAOR [*predicate(agent, object, recipient*)] forms. A narrative construction maps multiple sentences onto a situation model, specified as a network of these PAORs, linked by relations along the five dimensions of Zwaan and Radvansky (1998): time, space, causation, motivation, and protagonist. The narrative construction is compositional, built up from multiple sentences that are linked by relations along these dimensions. The nature of such relations and their representation has been identified in various discourse models, such as Centering Theory (Grosz and Sidner, 1986; Grosz et al., 1995), rhetorical structure theory (Mann and Thompson, 1988), SDRT (Lascarides and Asher, 1993), or coherence and structure of discourse (Hobbs, 1985). Taking the analogy from grammatical constructions, these relations are coded by the order of the sentences and by narrative function words (e.g., *but, since, then, so, now, because*, etc.). The crucial notion is that narrative structure provides a higher level of organization upon the events that it describes. New links—causal, intentional, temporal, etc., and aspects of meaning about people and events that may breach the canonical structure—are superimposed on the raw events by the narrative discourse, and this structuring results in the creation of meaning referred to by Bruner (1990, 1991, 2009), that are addressed in detail below. This superposition of enriched meaning onto events is analogous to how grammatical structure provides a higher level of organization on an unorganized ensemble of words in a sentence. In this context, we can now elaborate our implementation approach:

1. Narrative comprehension and production shall be implemented by starting with the existing conception and implementation of the grammatical construction and extending it to a novel formulation of narrative construction.
2. Analogous to the way that sentence-level constellations of grammatical function words specify thematic role relations between words in a sentence, constellations of narrative function words in a narrative specify the relations across sentences and events, represented in the situation model (SM).
3. In the meaning pole of the narrative construction, in addition to the representation of the current PAOR event, there will be a new context component that allows representation of the narrative links as specified by the narrative function words. This contributes to the situation model (SM) which represents the connected events of the narrative via these narrative links.

As noted already, we have developed methods for representing and expressing meaning about physical events in grammatical constructions (Dominey and Boucher, 2005; Hinaut et al., 2014). The constructions are learned by observation of how humans communicate such meaning in sentences. That is, paired <sentence, meaning> corpora are created, and used to train the comprehension and production models. This notion is now extended to narrative constructions, which allow humans to communicate meaning about a group of events that occurred in a coherent behavioral context, and importantly to express relations between events that may not be visible. If the grammatical construction uses word order and grammatical functions words to map open class elements onto their thematic roles, then the narrative construction uses sentence order and narrative function words to map multiple sentences onto events and relations between them. The form pole of the narrative construction is thus composed of a sequence of sentences that are linked via narrative function words—much like the grammatical function words (closed class words) that provide the grammatical structure at the sentence level.

This requirement for the existence of narrative function words is born out in the existence of discourse connectives or narrative function words—which provide discourse structure (Grosz and Sidner, 1986; Knott, 1996; Knott and Sanders, 1998; Fraser, 1999; Webber et al., 2001), much like grammatical function words (closed class words) provide grammatical structure at the sentence level. Interestingly, Norrick (2001) shows how discourse markers “well” and “but” can take on special narrative functions distinct from their lexical meanings and usual discourse marker functions. This contributes to the argument for the predicted psychological validity of the notion of narrative function word. Again, there is no claim that inserting words like “well” and “but” make a set of sentences into a narrative, any more than inserting “to” and “by” into a set of words makes them into a grammatical construction or sentence. Rather the claim is that words like “well” and “but” can provide extra meaning that contributes to causal and temporal linkages between events in narrative structure.

Narrative constructions are thus learned as conventions, in the same way that grammatical constructions are learned as conventions. As with the grammatical construction model, the system must be furnished with matched sentence-meaning pairs. The novelty is that these sentences will include narrative function words, whose role will also be reflected in the meaning representation. That is, they will be intrinsically present in the sequential structure of sentences and in the meaning representations in training corpora, and learned by the system. Crucially, however, as mentioned above, there may be components of the narrative structure that are not visible in the physical events, e.g., causal and logical relations. These relations will be introduced by the narrator in the narrative examples. This is part of how narrative is used to make meaning (Bruner, 1990, 1991).

### Learning a simple narrative

The first exercise is to determine if indeed the narrative production and comprehension models can learn to produce and understand narratives based on a corpus with the characteristics illustrated in Table 1. Comprehension will be tested by providing the model with this corpus of <sentence:context:meaning> triplets, and validating that when given a narrative with the same format, the system can generate the corresponding context and meaning. In the opposite sense, for production, the production model will be trained with the same corpus, and then tested by providing the context and meaning, and determining whether it can produce the correct narrative output. The proof of concept will use the corpus that is detailed in Table 1 both for production and comprehension. As illustrated in Table 1 each sentence is a well-formed English sentence, and the meaning is coded in a predicate argument representation, and the focus hierarchy. Recall that as described above, the focus hierarchy enumerates the semantic roles of the successive open class elements, thus specifying the hierarchy of focus in terms of order. Importantly, the meaning component also includes the narrative context. This is made up of the narrative function words and their mapping into the sentence. This additional information corresponds to the Narrative Relations in Figure 1.

**Table 1:** Form and meaning poles of a set of sentences making up a narrative construction.

**Meaning**		**Form**
**Context:**		
**Open class words in roles: Predicate agent object recipient**	**Focus hierarchy**	**Narrative sentence**
,	[_-_-_-_-_-_-_-_]	
wanted I, get I giraffe	[A-P-_-_-_-_-_-_] [A-_-P-O-_-_-_-_]	I wanted to get the giraffe
but,	[P-_-_-_-_-_-_-_]	
failed I, grasp I it	[_-A-P-_-_-_-_-_] [_-A-_-P-O-_-_-_]	but I failed to grasp it
because,	[P-_-_-_-_-_-_-_]	
laid it outofreach	[_-A-P-R-_-_-_-_]	because it laid outofreach
so,	[P-_-_-_-_-_-_-_]	
found I action different	[_-A-P-R-O-_-_-_]	so I found a different action
if,	[P-_-_-_-_-_-_-_]	
could I, ask I you, give you it me	[_-A-P-_-_-_-_-_] [_-A-_-P-R-_-_-_] [_-_-_-_-A-P-O-R]	if I could ask you to give it to me
then,	[P-_-_-_-_-_-_-_]	
would you, give you it me	[_-A-P-_-_-_-_-_] [_-A-_-P-O-R-_-_]	then you would give it to me
so,	[P-_-_-_-_-_-_-_]	
asked I you, give you it me	[_-A-P-R-_-_-_-_] [_-_-_-A-P-O-R-_]	so I asked you to give it to me
and,	[P-A-_-_-_-_-_-_]	
gave you it me	[_-_-A-P-O-R-_-_]	and you gave it to me
now because,	[_-_-_-P-A-_-_-_-_-_]	
have I giraffe, gave you it me	[A-P-O-_-_-_-_-_-_] [_-_-_-V-_-A-P-O-R]	I have the giraffe now because you gave it to me

*The form pole corresponds to the sentences making up the narrative. The meaning pole consists of the semantic words in their predicate, agent, object, recipient frames, and the corresponding focus hierarchy which indicates the position of these elements in the sentence frame. The narrative context component of the meaning pole (shaded in gray) indicates the narrative semantic words, (left column) and their position in the sentence frame (right/middle column). Note that these narrative context elements do not refer to any specific event, but rather to links between events, or intentional states. The segment “but I failed to grasp it” consists of its basic event, corresponding to “I failed to grasp it,” and the discourse component “but.” The basic event is coded as two linked predicates failed(I), grasp(I, it), represented in the left column. In the middle column is the mapping of these semantic elements into the phrasal semantic structure of the sentence. This explicitly maps the semantic roles into the positions of the semantic words in the sentence. The gray shaded row provides this same representation for the discourse context component*.

**Simulation Test 1—**Objective: Verify that the system can extract meaning in terms of events, and narrative relations, from a narrative. The comprehension model was tested on the corpus in Table 1 with 800 neurons in the reservoir. During training, the model was exposed to the form pole (the sentences) and the meaning pole (the phrasal semantics). Then in testing we presented just the sentences, and the model was required to reconstruct the corresponding phrasal semantics, including the component corresponding to the narrative function words. For each of the component constructions in this narrative construction, the model was able to successfully generate the meaning, corresponding to the first two columns in Table 1.

**Simulation Test 2—**Objective: Verify that the system can generate a narrative from a meaning specified in terms of events, and narrative relations, Likewise, for narrative generation, the narrative production model with 800 neurons in the reservoir was tested on the corpus from Table 1. During training, the model was exposed to the form pole (the sentences) and the meaning pole (the phrasal semantics). Then in testing we presented just the phrasal semantics, and the model was required to reconstruct the corresponding sentence that expressed that phrasal semantics, including the narrative function words. For each of the component construction in this narrative construction, the model was able to successfully generate the sentences, corresponding to the third column in Table 1.

### Metaphorical transfer by argument substitution

Recall that the grammatical construction model allows an open set of sentences to be generated from a single construction, by substituting new semantic words in the arguments. Similarly so for narrative, and this power of argument substitution will be much greater at the level of the narrative. Lakoff and colleagues have developed extensive arguments that human meaning is grounded in metaphorical mapping to sensorimotor experience (Lakoff and Johnson, 2008; Lakoff and Narayanan, 2010). A model of construction processing should ideally be able to account for this. Interestingly, because narrative constructions must take open class words (semantic words) as their arguments, these constructions can serve as an explicit vehicle for metaphor.

For example, consider the sentence: “I understand math now because you explained it to me” compared with the earlier sentence “I have the giraffe now because you gave it to me.” One can see that the causal relation between “have” and “gave” is generalized to create this relation between “explain” and “understand.”

That is, at the level of the single construction, understand-explained will inherit the causal link associated explicitly with have-gave, via the narrative function word “because” which has become associated with this mapping. Thus, the sentence “I understand …” inherits the causal structure from the sentence “I have …”. If we extend this mapping, that is, map have-understand, giraffe-math, gave-explained, we can then look at argument generalization and structure inheritance at the level of the consolidated narrative construction.

**Simulation Test 3**—Objective**:** Verify that the system can generate a narrative from a meaning using arguments that were not present in the learned corpus. The narrative production model is tested in the same conditions as described above, substituting the open class words as specified in Table 2. This simulation automatically generated the following narrative:

*I wanted to get the math, but I failed to grasp it, because it laid outofreach. So I found a different action. If I could ask you to explain it to me, then you would explain it to me. So I asked you to explain it to me, and you explained it to me. I understand now the math. I understand the math now because you explained it to me. You explained me the math because I wanted it*.

The meaning inherent in the narrative construction, e.g., the causal links between the intentional state of the narrator (wanting) and the resulting events in the discourse, are inherited into new event ensembles, and thus used to impose meaning upon them. The narrative construction is a meaning generator: it allows the listener to realize that explaining is a way of giving, and that there is a meaningful causal link between wanting and explaining.

**Table 2:** Semantic words in the giraffe complication-resolution scenario, and their replacements in the new “math” scenario.

Giraffe scenario arguments	Giraffe	Have	Give/gave
Mapping to math scenario arguments	Math	Understand	Explain/explained

### Narrative constructions for analogical transfer

The notion of construction leveraged in this work provides a framework for understanding two scenarios as sharing an underlying structure. Lakoff has extensively developed this notion of shared structure in the concept of metaphor as a mode of thought, defined by a systematic mapping from a source to a target domain (Lakoff, 2006). He identifies three characteristics of metaphor use:

1. The systematicity in the linguistic correspondences.
2. The use of metaphor to govern reasoning and behavior based on that reasoning.
3. The possibility for understanding novel extensions in terms of the conventional correspondences.

Our example of argument substitution meets these criteria, and illustrates an example of how narrative structure could generate meaning in a novel context, introducing a causal role for intentions, and the notion that explaining is a force and understanding a consequential result. Here we try to extend this notion of metaphor into the domain of analogical problem solving (Gick and Holyoak, 1983). In a hallmark paper, Gick and Holyoak explored the process of mapping across subject domains in analogical problem solving, using the “radiation problem” initially introduced by Dunker (Duncker and Lees, 1945). In this problem, a doctor has a patient who has a malignant tumor in his stomach. It is impossible to operate, but the tumor must be destroyed. There is a kind of ray that can be used to destroy the tumor, but at the appropriate intensity to destroy the tumor, the ray will also destroy the tissue it passes through. At lower intensities the rays are harmless to the tissue, but also to the tumor. What kind of procedure could be used to destroy the tumor with the rays, without destroying the surrounding tissue?

Subjects who had previously been exposed to analogous “convergence schema” problems, were able, under certain circumstances, to use this schema to solve the problem. The solution involves using several low intensity rays that converge at the location of the tumor, thus destroying it while leaving the surrounding tissue unharmed. In their experiments, Gick and Holyoak developed several variants on the radiation problem. In the original problem, the doctor divides the ray into multiple converging pathways to the tumor. In the General problem, the general divides his army over multiple narrow streets to capture the enemy fortress, and in the Fire-chief problem, the fire-chief sends retardant foam through multiple small hoses so enough foam can extinguish the fire.

Once the convergence schema and the mapping between these three stories has been made explicit, it seems obvious how subjects could use knowledge of the convergence schema to solve the problem. Interestingly, the conditions in which this transfer actually occurred were quite particular. The key condition for subjects to robustly apply the analogy was to have read two example analogs, and to have been given a verbal or graphic depiction of the convergence schema principal after each story. Under these conditions, subjects came up with better representations of the convergence schema, and found the solution to the new problem significantly more often. The verbal principal that was presented after the “General” discourse was “The General attributed his success to an important principle: If you need a large force to accomplish some purpose, but are prevented from applying such a force directly, many smaller forces applied simultaneously from different directions may work just as well.” After the second story, the principal was worded exactly the same as for the General story, but with appropriate argument substitution.

Importantly, thus, they found that amongst the most reliable conditions for promoting analogical problem solving is when multiple examples are summarized in a standard format that captures the essence of the convergence schema, with exactly the same structure, and the specific elements of the each specific discourse supplied as arguments. In this context, we were able to create such a narrative for the doctor story that captures the analogical schema, and is then used to generate the solution to the General and the fire-chief problems.

**Simulation Test 4—**Objective: Verify that the system can learn an analogical schema and then apply it to generate multiple narratives that fit the analogy. The training corpus for the “convergence schema” construction is illustrated in Table 3, and the set of argument substitutions for the doctor, general and fire-chief stories are presented in Table 4. Using the production model with 800 reservoir neurons, and the arguments corresponding to the doctor version of the convergence schema, the following discourse was produced (see Box 1).

**Table 3:** Narrative construction implementing the analogical convergence schema from Gick and Holyoak.

**Meaning**		**Form**
**Context:**		
**And Open class words in roles: Predicate agent object recipient**	**Focus hierarchy**	**Narrative sentence**
,	[_-_-_-_-_]	
had AGENT VICTIM DANGER LOCATION	[A-P-O-R-V] [_-_-_-_-_]	The AGENT had a VICTIM with a DANGER in the LOCATION.
could,	[_-_-_-P-_-_-_-_]	
has AGENT TOPIC, eliminate TOPIC DANGER LOCATION	[A-P-O-_-_-_-_-_] [_-_-A-_-P-O-_-R]	The AGENT has TOPIC that could eliminate the DANGER from the LOCATION.
enough,	[_-_-_-P-_-_-_-_-_]	
is TOPIC QUALITY, ACTION TOPIC DANGER	[A-P-O-_-_-_-_-_-_] [A-_-_-_-P-O-_-_-_]	The TOPIC is QUALITY enough to ACTION the DANGER.
if then,	[P-_-_-_-_-A-_-_-_]	
reach TOPIC DANGER simultaneously, destroy it DANGER	[_-A-P-O-R-_-_-_-_] [_-_-_-_-_-_-A-P-O]	If the TOPIC reaches the DANGER simultaneously then it destroys the DANGER.
unfortunately enough,	[P-_-_-_-_-_-_-_-_]	
is VECTOR CONSTRAINT too, reach TOPIC LOCATION simultaneously	[_-A-P-O-R-_-_-_-_] [_-_-_-_-_-A-P-O-R]	Unfortunately the VECTOR is too CONSTRAINT for the TOPIC to reach the LOCATION simultaneously.
cannot,	[_-_-_-_-_-P-_-_-_]	
arrival TOPIC limited, CONSTRAINT VECTOR, ACTION DANGER	[O-P-A-_-_-_-_-_-_] [_-_-_-P-A-_-_-_-_] [_-_-_-_-_-_-A-P-_]	Limited arrival of the TOPIC by the CONSTRAINT VECTOR cannot ACTION the DANGER.
fortunately,	[P-_-_-_-_-_-_-_-_]	
developed AGENT solution novel	[_-A-P-O-R-_-_-_-_]	fortunately the AGENT developed a novel solution.
,	[_-_-_-_-_-_-_-_-_]	
send TOPIC VECTOR CONSTRAINT, allow convergence, ACTION TOPIC DANGER	[P-A-R-O-_-_-_-_-_] [_-_-_-_-P-O-_-_-_] [_-A-_-_-_-_-P-O-_]	sending TOPIC on CONSTRAINT VECTOR allows convergence to ACTION the DANGER
thus,	[_-P-_-_-_-_-_-_-_]	
divides AGENT TOPIC, Multiple CONSTRAINT VECTOR	[A-_-P-O-_-_-_-_-_] [_-_-_-_-P-A-O-_-_]	The AGENT thus divides the TOPIC over multiple CONSTRANT VECTORS
so,	[P-_-_-_-_-_-_-_-_]	
converged TOPIC, ACTION TOPIC DANGER, save TOPIC VICTIM DANGER	[_-A-P-_-_-_-_-_-_] [_-A-_-P-O-_-_-_-_] [_-A-_-_-_-P-O-R-_]	so the TOPIC converged to ACTION the DANGER and save the VICTIM from the DANGER.

**Table 4:** Mapping of arguments in the three convergence problems onto the convergence schema narrative construction.

	**Agent**	**Victim**	**Danger**	**Location**	**Topic**	**Quality**	**Action**	**Vector**	**Constraint**
1	Doctor	Patient	Tumor	Thorax	Radiation	Strong	Kill	Flesh	Fragile
2	General	Town	Enemy	Fortress	Army	Large	Capture	Roads	Narrow
3	Fire-chief	Oil-well	Fire	Source	Foam	Retardant	extinguish	Hoses	Small

Box 1 **Discourse produced by narrative production model and narrative construction in Table 3 and arguments from Table 4 for the doctor problem.**

The doctor had a patient with a tumor in the stomach. The doctor has a ray that could eliminate the tumor from the stomach. The ray is strong enough to destroy the tumor if the ray reach the tumor simultaneously then it destroys the tumor. Unfortunately the flesh is too fragile for the ray to simultaneously reach the stomach. Limited arrival of the ray by the fragile flesh cannot destroy the tumor. Fortunately the doctor developed a novel solution. Sending the ray on the fragile flesh allows convergence to destroy the tumor. The doctor thus divides the ray over multiple fragile flesh. So the ray converged to destroy the tumor and save the patient from the tumor.

Once the narrative structure is learned for the doctor problem, this construction is used as an analogical schema corresponding to the convergence schema. The cognitive challenge is to find the appropriate mapping for elements from a new problem onto this schema. Then, as illustrated in Table 4, when the problems for the General with the enemy in the fortress of the town, or the fire-chief, with the fire in the source of the oil-well are presented, they are solved by inserting these arguments into the analogical schema that was initially developed in the context of the doctor problem.

The narrative construction for the doctor problem thus serves as an analogical schema for the more general convergence schema. By argument substitution, using the arguments in Table 4, we can exploit the narrative construction to find the solution to the general and fire-chief problems. The resulting narratives produced by the NCx production model are presented in Boxes 2, 3.

Box 2 **Discourse produced by narrative production model and narrative construction in Table 3 and arguments from Table 4 for the general problem.**

The general had a town with a enemy in the fortress. The general has a army that could eliminate the enemy from the fortress. The army is large enough to capture the enemy. If the army reach the enemy simultaneously then it destroy the enemy. Unfortunately the roads is too narrow for the army to simultaneously reach the fortress. Limited arrival of the army by the narrow roads cannot capture the enemy. Fortunately the general developed a novel solution. Sending the army on the narrow roads allows convergence to capture the enemy. The general thus divides the army over multiple narrow roads. So the army converged to capture the enemy and save the town from the enemy.

Box 3 **Discourse produced by narrative production model and narrative construction in Table 3 and arguments from Table 4 for the fire-chief problem.**

The fire-chief had a oil-well with a fire in the source. The fire-chief has a foam that could eliminate the fire from the source. The foam is retardant enough to extinguish the fire. If the foam reach the fire simultaneously then it destroy the fire. Unfortunately the hoses is too small for the foam to simultaneously reach the source. Limited arrival of the foam by the small hoses cannot extinguish the fire. Fortunately the fire-chief developed a novel solution. Sending the foam on the small hoses allows convergence to extinguish the fire. The fire-chief thus divides the foam over multiple small hoses. So the foam converged to extinguish the fire and save the oil-well from the fire.

The narrative construction has been created as a set of grammatical constructions modified to include narrative links and context, corresponding to the form-meaning mappings for the convergence schema. The narrative comprehension and production models were demonstrated to be able to learn this narrative construction. By substituting arguments, it was demonstrated that the narrative construction for the analogical schema could be used to generate solutions to the analogous doctor, general, and fire-chief problems. This suggests how language can provide a framework for the construal of meaning in the service of analogical reasoning (Gick and Holyoak, 1983; Lakoff, 2006; Lakoff and Johnson, 2008).

## Embodying the model in a cognitive systems framework

Here we demonstrate how this narrative learning capability is integrated in a robotic cognitive system, where the functional elements in Figure 1, the ABM, Situation Model, etc. are implemented and used for narrative learning in a human-robot interaction context. There are two principal motivations for this. First, using narrative in a developmental manner to allow the robot to construct and enrich meaning, analogous to the way that this is done in humans (Bruner, 1990; Nelson and Fivush, 2004) will make significant progress in the development of robot cognition and the understanding of human cognition. Second, in the more general study of narrative processing, this robotic application is of interest because the robot has knowledge of its own actions and so this knowledge helps to partially solve the problem of generating labeled corpora. Indeed, in the beginning, the robot will have a stored record of the actions it has performed, but will not necessarily have the accompanying text to communicate them, which will be provided by the human, analogous to how adults provide narrative structure to children that enriches the experience. Together the experience and the paired narrative are used to automatically generate labeled data for training the models.

Crucially, embedding the narrative construction system in the embodied robot context forces us to address issues of how meaning and language interact in an developmental context. The context of this work has been developed around the iCub, a 53 degree of freedom research robot created by the Italian Institute of Technology as part of the FP6 European project, RobotCub (Metta et al., 2010). Behavioral scenarios involve cooperative human-robot interaction with the iCub, including asking for help from the human when actions fail (Lallée et al., 2012, 2013; Petit et al., 2013; Pointeau et al., 2013, 2014; Hinaut et al., 2014). In our recent research, all of these events are encoded in an episodic memory which is part of an autobiographical memory (ABM) system (Pointeau et al., 2014). We now take advantage of this infrastructure so that following a given interaction, the human provides a narrative that describes the scenario and enriches the meaning. By matching arguments that appear in the narrative, with corresponding labels in the ABM, the system identifies the remembered events that the narrative describes, and creates a corpus of matched <sentence:meaning:context> triplets that are then used to train the comprehension and production models.

### Functional modules

As illustrated in Figure 1, the narrative construction model is situated in a system where it sits at the junction between the situation model and the narrative. Here we describe the components that make up the system.

#### Autobiographical memory (ABM)

At the core of the system is an autobiographical memory (ABM). The ABM is a structured set of SQL tables (see examples in Tables 5–**7**) and C++ coordination programs that encodes the interaction history of the robot, including everything it says and does, hears and observes (Pointeau et al., 2013, 2014). This provides the content for the situation model, which interacts with the narrative construction processing models. The system also implements a form of contextual or working memory, as a real-time snapshot of the current state of affairs, and the most recent semantic representations including the most recently perceived action, agent, object, and recipient. This will contribute to co-reference resolution.

**Table 5:** Principal SQL table of the ABM, regrouping all the events in the given period of execution of the human-robot interaction involving the complication-resolution scenario, where the iCub tries to grasp an object, fails, and asks the human for help.

**Idactivity**	**Time**	**Activityname**	**Activitytype**	**Instance**	**Begin**
20596	2016-05-20 16:53:02.560945	take	action	19061	TRUE
20597	2016-05-20 16:53:02.598254	take	action	19062	FALSE
20598	2016-05-20 16:53:05.038196	reason	reasoning	19063	TRUE
20599	2016-05-20 16:53:06.355905	reason	reasoning	19064	FALSE
20600	2016-05-20 16:53:08.765083	reason	reasoning	19065	TRUE
20601	2016-05-20 16:53:11.083612	reason	reasoning	19066	FALSE
20602	2016-05-20 16:53:12.705282	sentence	recog	19067	TRUE
20603	2016-05-20 16:53:14.54652	give	action	19068	TRUE
20604	2016-05-20 16:53:22.148949	give	action	19069	FALSE

*Instance number is the principal key for linking to other tables. This will contribute to the meaning representation for in the Situation Model*.

#### Situation model

The situation model serves as the interface between language and meaning. This is necessary, precisely because in the veridical record of experience in the ABM, there are levels of meaning that are not represented, and thus that cannot influence or be influenced by language. For example, “I gave you the giraffe because you wanted it” establishes a causal link between a mental state and an action that is not present in the ABM. The situation model allows for this level of representation (Zwaan et al., 1995; Zwaan and Radvansky, 1998; Zwaan and Madden, 2004; Madden and Dijkstra, 2010).

The situation model should allow access to events so that they can be linked to elements in the narrative. It should allow access to the context or state in which the event takes place, any goal associated with the event, temporal order, and the possibility to introduce causal (and other) relational links between events. We implemented a C++ data structure that satisfies these requirements, including the following fields: Initial state, Goal, Action, Result, Final state (IGARF). The Initial State encodes any relations that are active in the ABM at the onset of the action (Table 6—a secondary table in the ABM SQL database), the Goal specifies the desired relational states (typically not specified in the ABM), the Action is the atomic action specified in the ABM, the Result is the atomic outcome specified in the ABM. The final state is the set of relations that hold after the action (a secondary table in the ABM). The structure of an event is thus characterized by two action components: the Action and the Result, and three state components, initial state, goal state and final state. The resulting data structure is referred to as an IGARF for Initial, Goal, Action, Result, and Final stea. An example of a situation model is illustrated in Figure 2. Tables 5–7 illustrate the contents of the ABM that were generated during an interaction corresponding to the complication-resolution scenario. The situation model is generated automatically from these ABM tables (described below).

**Table 6:** Secondary table specifying relations that will be used to create the Situation Model.

**Instance**	**Subject**	**Verb**	**Object**
19061	Interlocutor	has	croco
19061	iCub	want	croco
19062	Interlocutor	has	croco
19062	iCub	want	croco
19063	Interlocutor	has	croco
19063	iCub	want	croco
19064	Interlocutor	has	croco
19064	iCub	want	croco
19065	Interlocutor	has	croco
19065	iCub	want	croco
19066	Interlocutor	has	croco
19066	iCub	want	croco
19067	Interlocutor	has	croco
19067	iCub	want	croco
19068	Interlocutor	has	croco
19068	iCub	want	croco
19069	iCub	has	croco

**Table 7:** Argument content table.

**Instance**	**Argument**	**Type**	**Subtype**	**Role**
19061	iCub	entity	agent	agent
19061	croco	entity	object	object
19061	take	external	default	predicate
19061	qRM	external	default	provider
19062	iCub	entity	agent	agent
19062	croco	entity	object	object
19062	take	external	default	predicate
19062	qRM	external	default	provider
19062	outofreach	external	default	reason
19062	failed	external	default	status
19063	iCub	entity	agent	agent
19063	(predicate have) (agent icub) (object croco)	external	default	goal
19063	reason	external	default	predicate
19063	abmReasoning	external	default	provider
19064	iCub	entity	agent	agent
19064	(predicate have) (agent icub) (object croco)	external	default	goal
19064	addressee#have#object	external	default	needs
19064	reason	external	default	predicate
19064	abmReasoning	external	default	provider
19064	(predicate sentence) (speaker icub) (object croco)	external	default	result
19065	iCub	entity	agent	agent
19065	reason	external	default	predicate
19065	abmReasoning	external	default	provider
19065	give	external	default	whatIs
19066	recipient#have#object	external	default	action_after
19066	agent#have#object	external	default	action_before
19066	iCub	entity	agent	agent
19066	reason	external	default	predicate
19066	abmReasoning	external	default	provider
19066	speaker#have#object	external	default	sentence_after
19066	addressee#have#object	external	default	sentance_before
19066	give	external	default	whatIs
19067	Interlocutor	entity	agent	addressee
19067	iCub	entity	agent	adj1
19067	Interlocutor	entity	agent	agent
19067	croco	entity	object	object
19067	give	external	default	predicate
19067	Give me the croco please	external	default	sentence
19067	iCub	entity	agent	speaker
19067	none	external	default	subject
19068	Interlocutor	entity	agent	agent
19068	croco	entity	object	object
19068	give	external	default	predicate
19068	iCub	entity	agent	recipient
19069	Interlocutor	entity	agent	agent
19069	croco	entity	object	object
19069	give	external	default	predicate
19069	iCub	entity	agent	recipient

*This contains complimentary information for events, in particular the crucial arguments: predicate, agent, object, recipient, and status that will make up the meaning in the Situation Model*.

#### Narrative construction model

The reservoir NCx models are the third part of the core of the system. They use an inventory of form-to-meaning mappings in order to go back and forth between representations of meaning and sentences (Hinaut et al., 2014). As described above, the novelty with respect to the reservoir models that were developed for isolated sentence comprehension and production is that these reservoirs manipulate narrative context, which allows relations to be expressed that link events across multiple sentences. The sentence-meaning corpora for training the models are generated from the robot's interaction: meaning is coded as the experience in ABM and SM, and sentences are provided by the human, who narrates the robot's behavior. In doing so, the human actually begins to impose narrative structure on the internal representation, and thus begins to allow the robot to create meaning that enriches its experience.

### Experimental validation

Here a complete execution of the system is described, starting with the contents of the ABM that encode the events derived from the complication-resolution scenario with the iCub and a human. The contents of the primary and secondary tables of the ABM are seen in Tables 5–7. The following four paragraphs correspond to the functions labeled 1–4 in Figure 1.

#### Autobiographical memory to situation model (ABMtoSM)

ABMtoSM is a C++ function that reads the contents of the ABM in a given time period, and automatically generates a situation model. Constructing the Initial state, Goal, Action, Result, Final state (IGARF) representations of events in the situation model from the ABM is trivial because the ABM contents map directly onto the IGARF, though not all of the information may be available. There are three possibilities as the beginning and the end of the action are specified allowing specification of the Initial and Final states, and the Result. (1) If the Result is a success and the Initial and Final states are different, then the Goal is the difference between the two. (2) If the result is a failure, then the Goal unspecified. (3) If only the onset of an action is known, then the Initial state and Action are specified, and the other fields are left unspecified. The Situation Model resulting from this function is displayed in Figure 2.

#### Situation model to training corpus (SMtoCorpus)

**Figure 5:**
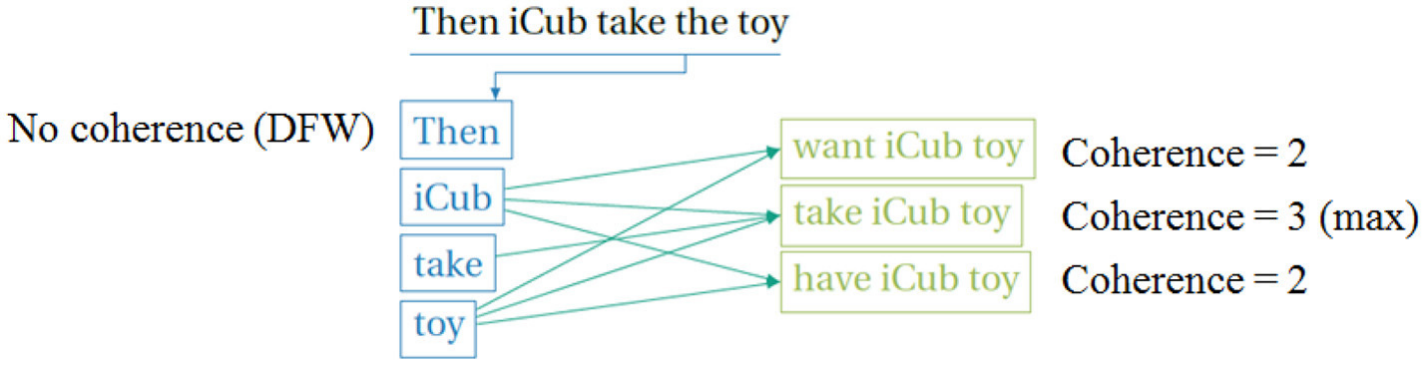
Calculating coherence between sentence in narrative and meanings in Situation Model during creation of the training corpus for the Narrative Construction reservoir models. To match the sentence with the appropriate meaning we calculate coherence as the number of matches between open class words in the sentence, and in the candidate meanings. The meaning atom with maximum coherence is selected as the meaning corresponding to the sentence. Words that have no match in the meaning are candidate narrative function words. DFW, discourse function word or narrative function word.

SMtoCorpus is a C++ function that reads the situation model, and the sentences in the narration, and automatically generates a training corpus that can be used by the NCx comprehension and production models. Recall that in the behavioral paradigm, the narrative that is provided by the human is able to enrich the mental representation. Before this can take place, however, there must be sufficient language acquisition that the system (child) can associate the narrative sentences with the corresponding meanings. This is implemented in a procedure that automatically generates a training corpus from the Situation Model and the matched narrative. For each sentence in the narrative, the SMtoCorpus module calculates the coherence between the sentence and each of the meaning atoms in the Situation Model, in order to find the meaning component that best corresponds to each sentence. This is schematically illustrated in Figure 5. For each sentence and meaning pair, the training corpus must also have the deterministic specification of the focus hierarchy, which is calculated directly from the sentence and meaning. Here is a simple narrative that is used for generating the corpus:

First iCub wants the croco, but when he grasps it, he fails to take, because it was out-of-reach. iCub reasons, and he thus says Give_me_the_croco_please to Sam, then Sam gave the croco to iCub. now iCub has the croco.

The training corpus that was generated automatically is illustrated in Table 8. Note that the system was able to detect the narrative function words and specify them at the start of each meaning atom specification. This means that these narrative function words will be available for enriching the meaning representation in the Situation Model. Note that pronouns can be used. A simple pronoun resolution system was implemented where subject pronouns (e.g., he, she) are mapped onto the agent role, object personal pronouns (e.g., him, her) on to the recipient role, and object impersonal pronouns (it) onto the object role. Synonyms were processed in a similar manner—a dictionary of synonyms was created and used during the matching process, so that words like “take” and “grasp” could be used as synonyms in the matching. Again, this is a proof of concept implementation. Once the training corpus is available, the comprehension and production networks are trained and complete the language interface with the situation model. Note that the narrative used here is relatively simple. This is because in the proof of concept for SMtoCorpus there must be a close correspondence between the ABM event description, and the contents of the sentences in the narrative that are matched with each event description. This limitation is a point to be addressed in the discussion of future work.

**Table 8:** Automatically generated corpus.

**Meaning**		**Form**
**Open class words: Predicate, agent, object, recipient**	**Focus**	**Sentence**
first,	[P-_-_-_-_-_-_-_]	
wants iCub croco	[_-A-P-O-_-_-_-_]	first iCub wants the croco
but when,	[P-A-_-_-_-_-_-_]	
grasps he it	[_-_-A-P-O-_-_-_]	but when he grasps it
fails he take	[A-P-O-_-_-_-_-_]	he fails to take
because,	[P-_-_-_-_-_-_-_]	
was it out-of-reach	[_-A-P-O-_-_-_-_]	because it was out-of-reach
reasons iCub	[A-P-_-_-_-_-_-_]	iCub reasons
and,	[P-_-_-_-_-_-_-_]	
says he Give_me … _please Sam	[_-A-P-O-R-_-_-_]	and he says Give_me … _please to Sam
thus,	[_-P-_-_-_-_-_-_]	
gives Sam croco iCub	[A-_-P-O-R-_-_-_]	Sam thus gives the croco to iCub
now,	[P-_-_-_-_-_-_-_]	
has iCub croco	[_-A-P-O-_-_-_-_]	now iCub has the croco

#### Narrative to situation model (NCx to SM)

NCxtoSM is a C++ function that reads the output of the NCx comprehension model, and maps it onto an existing situation model. This is the crucial interaction, where the narrative of the adult (human) allows the infant (robot) to enrich its representation of its experience. When the narrative that was used to train the comprehension model is then processed by the trained narrative comprehension model, the narrative links are used to enrich the situation model. The trained model will be able to extract the meaning atoms and the narrative function words for each sentence. This will allow enrichment of the Situation Model with narrative links. Narrative links are defined by three elements: the source meaning atom, the target meaning atom, and the linking word. Within the Situation Model, narrative links are represented in a table where each entry encodes the narrative function word, and a pointer to the source and target meaning atoms. The prototypical link of interest links actions with mental states, as in “Sam gave iCub the croco because iCub wanted it” because it specifies a causal relation that is not at all visible in the experience encoded in the ABM. The updated narrative links are illustrated in red in Figure 2.

#### Situation model to narrative (SMtoNCx)

SMtoNCw is a C++ function that creates input for the NCx production model from the Situation Model. Now that the training corpus has been created, the system can generate narrative from the contents of the situation model. There are two methods for this. The first and most reliable is to reuse the narrative construction verbatim, as we did in Section From Grammatical Construction to Narrative Construction. This produces a perfect copy of the narrative that was used in training. The second method performs a traversal of the SM in order to generate a sequence of sentences that produce the same meaning that was transmitted in the initial narrative. For a proof of concept implementation of how to perform this traversal, we exploit the table of narrative links described above. This means that in order to be included in the narrative, events must have at least one narrative link.

In addition to the meaning, the NCx production model requires the construal—that is the intended meaning and the focus hierarchy, or specification of the order in which the predicate, agent, object, and recipient should appear in the sentence. For English, there is a canonical order APOR, and when other information is not available, this is the default choice. Using the SM, the system is able to regenerate the narrative that was used in training, but this time directly from the SM and the table of narrative relations.

### End-to-end system

At the outset of this work it was a real challenge to imagine how, from an empty system, we could arrive at narrative processing for enrichment of a situation model based on the robot's experience. This section presented complete results on a proof of concept demonstration of the end-to-end function of the system. That is, based on experience from an interaction coded in ABM, the system automatically generated a Situation Model representation of this experience. Then, using a simple narrative of that experience, the system automatically generated a training corpus based on the matched sentence, meaning pairs from the narrative and Situation Model, respectively. This allowed the NCx comprehension model to process that same narrative, now extracting the narrative links and using them to enrich the situation model. This is the narrative construction of meaning that was our initial target (Bruner, 1990, 1991; Nelson, 2003).

**Figure 6:**
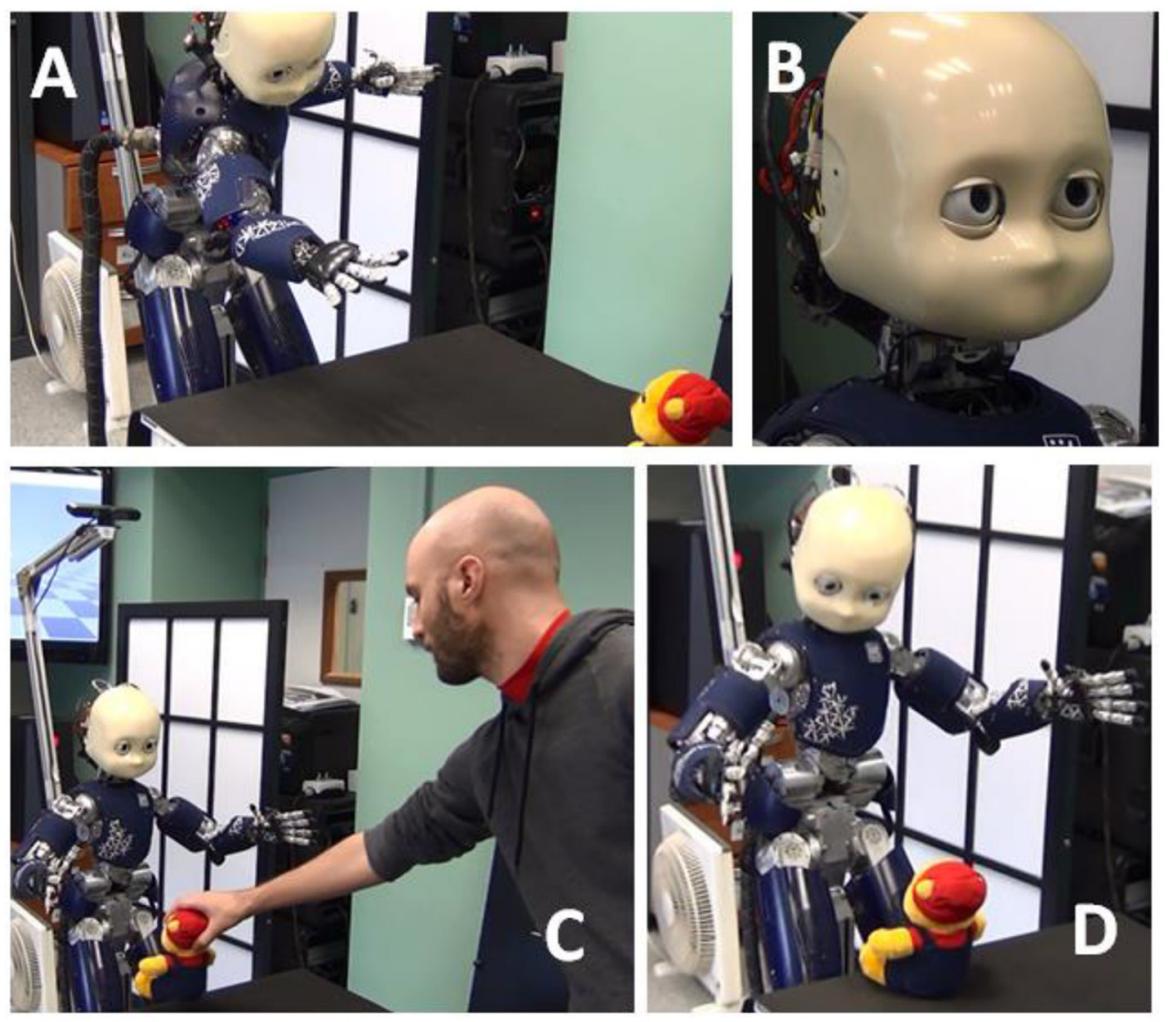
iCub interacting with the human. The iCub interacting with the human during the complication-resolution scenario. **(A)** iCub wants the Winnie and attempts to grasp it, and fails. **(B)** After identifying an alternative method to achieve its goal, iCub asks the human to give it the Winnie. **(C)** The human then gives the iCub the Winnie. **(D)** The goal is fulfilled, iCub has the Winnie. This interaction populates the autobiographical memory (ABM) providing the meaning pole for learning the narrative construction.

This illustrates how the system can function in five successive phases. In phase 1, the human-robot interaction behavior takes place, as illustrated in Figure 6. In phase 2, the robot provides a naïve description of events from the ABM. In phase 3, the human provides a more structured narrative that can be used to describe the same situation. This narrative is used to train the comprehension and production models. In phase 4 a new scenario is experienced, that is isomorphic in structure to the scenario in phase 1. Based on the learning of the narrative in phase 3, the same narrative construction can be applied, with argument substitution, to yield the new narrative in phase 5. These distinct phases can be observed in videos at the following link^1^. The resulting system accommodates narratives that increase the narrative dimension as exemplified in Table 9.

**Table 9:** Form and meaning poles of a set of sentences making up a narrative construction, augmented with sentences that increase the narrative dimension (indicated in *italic*).

**Meaning**		**Form**
**Context:**	
**And Open class words in roles: Predicate agent object recipient**	**Focus Hierarchy**	**Narrative Sentence**
,	[_-_-_-_-_-_-_-_]	
wanted I, get I giraffe	[A-P-_-_-_-_-_-_] [A-_-P-O-_-_-_-_]	I wanted to get the giraffe
*whenever previously*,	*[P-_-_-_-_-A-_-_-_]*	
*wanted I*, *get I something*, *was it easy*	*[_-A-P-_-_-_-_-_-_]* *[_-A-_-P-O-_-_-_-_]* *[_-_-_-_-_-_-A-P-O]*	*whenever I wanted to get something previously it was easy*
But now here,	[P-A-O-_-_-_-_-_-_-_]	
failed I, grasp I it	[_-_-_-A-P-_-_-_-_-_] [_-_-_-A-_-P-O-_-_-_]	but now here I failed to grasp it
because,	[P-_-_-_-_-_-_-_]	
laid it outofreach	[_-A-P-R-_-_-_-_]	because it laid outofreach
*never*,	*[_-_-_-_-_-P-_-_-_]*	
*seemed it like*, *would I*, *get I giraffe*	*[A-P-O-_-_-_-_-_-_]* *[_-_-_-P-A-_-_-_-_]* *[_-_-_-A-_-_-P-O-_]*	*it seemed like I would never get that giraffe*
*but*,	*[P-_-_-_-_-_-_-_]*	
*had I*, *have I it*	*[_-A-P-_-_-_-_-_]* *[_-A-_-P-O-_-_-_]*	*but I had to have it*
so,	[P-_-_-_-_-_-_-_]	
found I action different	[_-A-P-R-O-_-_-_]	so I found a different action
if,	[P-_-_-_-_-_-_-_]	
could I, ask I you, give you it me	[_-A-P-_-_-_-_-_] [_-A-_-P-R-_-_-_] [_-_-_-_-A-P-O-R-]	if I could ask you to give it to me
then,	[P-_-_-_-_-_-_-_]	
would you, give you it me	[_-A-P-_-_-_-_-_] [_-A-_-P-O-R-_-_]	then you would give it to me
so,	[P-_-_-_-_-_-_-_]	
asked I you, give you it me	[_-A-P-R-_-_-_-_] [_-_-_-A-P-O-R-_]	so I asked you to give it to me
,	[P-_-_-_-_-_-_-_]	
gave you it me	[_-A-P-O-R-_-_-_]	and you gave it to me
now because,	[_-_-_-P-A-_-_-_-_-_]	
have I giraffe, gave you it me	[A-P-O-_-_-_-_-_-_] [_-_-_-_-_-A-P-O-R]	I have the giraffe now because you gave it to me
because,	[_-_-_-_-P-_-_-_-_]	
gave you giraffe me, wanted I you	[A-P-R-O-_-_-_-_-_] [_-_-_-_-_-A-P-O-_]	you gave me the giraffe because I wanted you to
*again*,	*[_-_-_-P-_-_-_]*	
*is life good*	*[A-P-O-_-_-_-_]*	*life is good again*

## Cross-linguistic validation: direct application to Japanese

The current approach to language processing is based on the cue competition model, where cues including word order and grammatical morphology compete across languages to allow the coding of meaning (Bates et al., 1982, 1991; Bates and MacWhinney, 1987; Li and MacWhinney, 2013). In this context, one of the predictions of our model of narrative construction processing is that there should be no inherent difficulty in processing narrative across languages if they adhere to the cue competition. That is, the notion that narrative function words will establish links between meaning components in narrative should hold across languages. As in the previously demonstrated the cross-linguistic capabilities of our sentence comprehension (Dominey et al., 2006) and production (Hinaut et al., 2015) models, the narrative system is to be similarly validated.

**Simulation Tests 5 and 6**—A Japanese training corpus (Table 10) was first generated by hand and used to validate that the narrative production and comprehension models were capable of learning this corpus. The meaning of these sentences is clarified in Table 11. The NCx comprehension and production models, each with 800 neurons, were trained and tested on this corpus. In Simulation Test 5, the comprehension model successfully generated the meanings and narrative links from the narrative, and in Simulation Test 6, the production model successfully generated the narrative from the meanings and narrative links. In Japanese the grammatical function words are the particles that follow open class words (not complete): -wo (subject), -wa (object), -no (possessive), -ni (toward), -ga (subject). Narrative function words are (not complete): shikashi (but), node (so), dakara (and-so), you (because/in-order-to). This is interesting in that it extends the notion of narrative constructions, grammatical function words and narrative function words to Japanese. Importantly it also validates the cue-competition model for narrative constructions.

**Table 10:** Form and meaning poles of a set of sentences making up a narrative construction in Japanese.

**Meaning**		**Form**
**Context:**		
**And Open class words in roles: Predicate agent object recipient**	**Focus Hierarchy**	**Narrative Sentence**
,	[_-_-_-_-_-_-_-_]	
shitaidesu watashi,	[A-_-_-P-_-_-_-_]	
shutoku watashi wani	[A-O-P-_-_-_-_-_]	watashi wa wani wo shutoku shitaidesu
node,	[P-_-_-_-_-_-_-_]	
dekimasendeshita watashi,	[_-A-_-_-_-P-_-_]	
haaku_suru watashi sore	[_-A-O-P-_-_-_-_]	node watashi wa sore wo haaku_suru koto ga dekimasendeshita
dakara,	[P-_-_-_-_-_-_-_]	
mitsukemashita watashi dousa betsu	[_-A-R-O-P-_-_-_]	dakara watashi wa betsu no dousa wo mitsukemashita
,	[_-_-_-_-_-_-_-_]	
dekireba watashi,	[A-_-_-_-_-_-P-_]	
tanomu watashi,	[_-A-R-O-P-_-_-_]	
ataeru anata sore watashi	[_-A-R-O-P-_-_-_]	watashi wa anata ni watashi ni sore wo ataeru you ni tanomu koto ga dekireba
,	[_-_-_-_-_-_-_-_]	
darou anata,	[A-_-_-_-P-_-_-_]	
ataeru anata sore watashi	[A-R-O-P-_-_-_-_]	anata wa watashi ni sore wo ataeru darou
dakara,	[P-_-_-_-_-_-_-_]	
tanomu watashi,	[_-A-_-_-_-_-P-_]	
ataeru anata sore watashi	[_-_-R-O-P-A-_-_]	dakara watashi wa watashi ni sore wo ataeru you ni anata ni tanomu
to,	[P-_-_-_-_-_-_-_]	
ataemashita anata sore watashi	[_-A-R-O-P-_-_-_]	to anata wa watashi ni sore wo ataemashita
,	[_-_-_-_-_-_-_-_]	
tanonda watashi,	[_-A-_-_-_-P-_-_]	
ataeru anata sore watashi	[_-A-O-P-_-_-_-_]	watashi wa watashi ni sore wo ataeru you ni anata ni tanonda
node ima	[P-_-A-_-_-_-_-_]	
motte_imasu watashi wani	[_-A-_-O-P-_-_-_]	node watashi wa ima wani wo motte_imasu

**Table 11:** Correspondence between Japanese and English meaning.

Japanese	watashi wa	wani wo	shutoku_shitaidesu		
English	I-subject	croco-object	wanted to have		
Japanese	shikashi	sore wa	watashi_no_te_no_todokanai_tokoro	datta	
English	But	it-subject	place_where_my_hand_cant_go	was	
Japanese	node	watashi wa	haaku_suru koto ga	dekimasendeshita	
English	So	I-subject	take	failed	
Japanese	dakara	watashi wa	riyuu		
English	And_so	I-subject	reason		
Japanese	watashi wa	watashi_ni_wani_wo_ataeru you ni	anata ni	tanomu	
English	I-subject	Give_me_the_croco-object	you-recipient	ask	
Japanese	to	anata wa	watashi ni	wani wo	ataemashita
English	And	you-subject	me-recipient	croco-object	give
Japanese	watashi wa	watashi_ni_wani_wo_ataeru you ni	anata ni	tanonda	
English	I-subject	Give_me_the_croco-object	you-recipient	asked	
Japanese	node	watashi wa	ima	wani wo	motte_imasu
English	So	I-subject	now	croco-object	have

### Experimental validation of narrative enrichment in Japanese

The NCx models can be used for comprehension and production of Japanese narrative, by adding the Japanese particles to the list of grammatical function words recognized by the models. The next step is to verify that the enrichment of a situation model by narrative can function in Japanese. The Situation Model was first created from the ABM, as described above using ABMtoSM with the same SM as in the example above. A simple narrative was then created in Japanese, describing the complication-resolution scenario. To accommodate Japanese open class words, the synonym dictionary that is used in SMtoCorpus was updated, specifying the equivalence of the Japanese terms and the English terms that are used in the ABM (see Table 12). This way, the coherence could be calculated between the Japanese sentences and the ABM. This allowed execution of the Situation Model to Training Corpus transformation, and generation the Japanese corpus specified in Table 13.

**Table 12:** Synonyms.

have has	motte_imasu shutoku
take grasp	haaku_suru
say ask	tanomu tanonda
iCub icub	watashi
reason	riyuu
give	ataeru ataemashita
Sam Interlocutor	anata
croco	wani
want wants	shitaidesu shutoku_shitaidesu
fail fails	dekimasendeshita
is was	datta
Give_me_the_croco_please	watashi_ni_wani_wo_ataeru
out-of-reach	watashi_no_te_no_todokanai_tokoro
it	sore

**Table 13:** Automatically generated Japanese Corpus.

**Open-class words**	**Focus**	**Sentence**
shutoku_shitaidesu watashi wani	[A-O-P-_-_-_-_-_]	watashi wa wani wo shutoku_shitaidesu
shikashi,	[P-_-_-_-_-_-_-_]	
datta sore watashi_no_…tokoro	[_-A-O-P-_-_-_-_]	shikashi sore wa watashi_no…tokoro datta
node,	[P-_-_-_-_-_-_-_]	
dekimasendeshita watashi haaku_suru	[_-A-O-P-_-_-_-_]	node watashi wa haaku_suru koto ga dekimasendeshita
dakara,	[P-_-_-_-_-_-_-_]	
riyuu watashi	[_-A-P-_-_-_-_-_]	dakara watashi wa riyuu
		
dakara,	[P-_-_-_-_-_-_-_]	
tanomu watashi watashi_ni…ataeru anata	[_-A-O-R-P-_-_-_]	dakara watashi wa watashi_ni…ataeru you ni anata ni tanomu
to,	[_-A-R-O-P-_-_-_]	
ataemashita anata wani watashi	[_-A-R-O-P-_-_-_]	to anata wa watashi ni wani wo ataemashita
tanoda watashi watashi_ni…ataeru anata	[A-O-R-P-_-_-_-_]	watashi wa watashi_ni…ataeru you ni anata ni tanoda
node ima,	[_-A-_-O-P-_-_-_]	
motte_imasu watashi wani	[_-A-_-O-P-_-_-_]	node watashi wa ima wani wo motte_imasu
Open-Class words	Focus	Sentence
shutoku_shitaidesu watashi wani	[A-O-P-_-_-_-_-_]	watashi wa wani wo shutoku_shitaidesu

With this corpus, the SM could then be enriched with the narrative links specified by the narrative function words, using the Narrative to Situation Model module, which takes the output from that NCx comprehension model. The narrative links corresponding to those in the narrative in Table 13 were then automatically added to the situation model that was generated from the ABM. This completes the cross-linguistic validation of the system.

## Discussion

This research proposed a hypothesis and proof of concept demonstrations about the extension of the usage-based learning of grammatical constructions to narrative constructions. The existing models of grammatical construction processing for sentence comprehension and production were extended to models of narrative comprehension and production. The narrative construction, like the grammatical construction, is defined as a form-to-meaning mapping. The form pole is the collection of sentences that make up the narrative. The meaning pole corresponds to a set of predicate-argument representations of events that are linked by narrative relations in order to form the situation model. The notion of narrative construction thus represents a socially meaningful frame that enhances the meaning of a set of coordinated events, similar to the notion of pragmatic frame (Rohlfing et al., 2016).

The narrative comprehension and production models were demonstrated to learn to understand and produce narrative. Through argument replacement, the learned constructions were applied to understanding new narrative, and to generating new narrative. One of the most interesting results of this argument transfer is the ability associate the meaning of the word “because” with causal relations, and then to transfer this causal structure to intentional verbs like “want.” We thus provide a mechanism for describing how intentional roles are given to drives and intentions.

This attribution of intentional roles to mental states is a good illustration of the notion of narrative enrichment presented in the introduction. Throughout development, language enriches representations that are initially created through perception (Mandler, 2012; Nomikou et al., 2016). Through narrative enrichment, links along the five dimensions of Zwaan and Radvansky (1998) are introduced into the situation model, allowing relations that are invisible in the physical scene, such as the causal role of an intentional state, to become explicit. Once established, canonical or repetitive narrative constructions can be reused by substituting in new arguments.

The concept of argument substitution in narrative constructions is linked to the notions of metaphor and analogical transfer. A construction was learned in the context of a physical action for acquiring an object. We showed that by argument substitution, the notion of acquisition could be applied to knowledge that could be acquired via explaining vs. giving. This then led to the more extended idea of analogical schemas and analogical problem solving. This conception of narrative construction can serve at least as a framework or basis for the implementation of analogical schemas. This is consistent with Gick and Holyoak's observation that providing an explicit, structured narrative characterization of the convergence schema with two different argument sets (e.g., doctor and general) was amongst the most favorable conditions for subjects to use an analogical schema (Gick and Holyoak, 1983). Interestingly this also argues that the construction itself has meaning, independent of the arguments. This notion is foundational in the grammatical construction framework, and it has recently been demonstrated to have neurophysiological validity (van Dam and Desai, 2016).

We believe that this research makes a useful proposal and proof of concept of how narrative constructions can be learned and used, and how narrative can be used to enrich meaning. However, this work is by no means complete, and has a number of limitations and at the same time makes contact with existing data. A principal limitation of our usage-based learning method is that in order to be able to describe a situation, one must have already heard someone else describe that same (or isomorphic) situation. That is, narrative constructions must be learned before they can be used. Interestingly, this was the same criticism of our grammatical construction model. There are two responses to this criticism. The first is that indeed, young children's sentence production is highly conservative, using constructions they have already learned, and that have been heard in use by adults (Lieven et al., 2003). In other words, one can get a long way only using constructions that have been heard previously. The second response is that with a sufficiently large construction inventory (that is, with sufficient learned experience) the systems can robustly generalize to new constructions that were not present in the training (Hinaut and Dominey, 2013). We would thus extend this observation that we have made in the domain of grammatical constructions, into the domain of narrative constructions.

A related limitation or potential criticism of this work is related to the utility of the notion of the narrative construction as an extension of the grammatical construction. Since meaning, and the structuring and creation of meaning, is central to narrative, the notion of the construction as a form to meaning mapping should be relevant. In the grammatical construction we consider that, in an implementation of the cue competition hypothesis of Bates and MacWhinney, cues including word order and grammatical function words specify the form to meaning mapping. In the narrative construction we would extend this to include the notion of narrative function words.

The narrative construction thus builds on the grammatical construction, inheriting its functionality, and extending it with the crucial ability to build links across events, and thus create new meaning forms. With the narrative construction, a set of data generated by observing events can become structured into a meaningful and enriched whole. This notion of narrative construction thus serves as a format in which events can be organized into connected narrative, with higher order structure. The current demonstration can be considered a form of proof of concept, and future work must address more extended narrative structure in this narrative construction framework. The cross-linguistic validation with Japanese demonstrates an application of the cue competition hypothesis to narrative and a validation that it is robust across at least these two languages, English and Japanese.

The future work will take several directions. Experience with different forms of narrative, in play, in stories, and especially in talk about personal episodes, provides a model for organizing one's own episodic memories into the kind of narratives that emphasize personhood, motivations, goals, outcomes, emotions, and values (Nelson and Fivush, 2004). Narrative can be used to identify or create new relations that are not explicitly present in the system. This has been demonstrated with words (Waxman and Markow, 1995), and it is likely that the same is true for narrative. While it seems clear that narrative adds meaning beyond what is present in the raw events, one can ask about the relevance of this to discussions of the influence of language on thought. In a review of this question, Bloom and Keil (2001) consider that “Language may be useful in the same sense that vision is useful. It is a tool for the expression and storage of ideas. It is not a mechanism that gives rise to the capacity to generate and appreciate these ideas in the first place (p. 364).” This is in contrast with Bruner (1991) who proposes two distinct modes of thought. One is logical, rational, propositional, related to reasoning, and the search for truth conditions. The other is narrative, concerned with the search for meaning in experience. Narrative helps to endow experience with meaning. A crucial question then concerns how narrative is learned. We proposed an extension of the notion of grammatical construction to narrative constructions. The results presented here suggest that this notion of narrative construction may be of explanatory value. We will continue to address these questions in our future research with the narrative construction model in the context of the iCub and Pepper humanoids and their interaction with humans, encoded in autobiographical memory (Pointeau et al., 2014).

Likewise, the possible use of the narrative construction in the domain of metaphor and analogy will be investigated. In the context of metaphor, Feldman and Lakoff and the Berkeley Neural Theory of Language group consider that meaning is grounded in sensorimotor schemas which can be used through metaphor to understand more abstract meanings (Feldman, 2008; Lakoff, 2009). In this context, we consider that it must also be possible for new structures (in addition to the sensorimotor image schemas or conceptual schemas) to be learned in order to capture narrative structure. This is the narrative construction. We demonstrate that the adaptive narrative construction can be learned to accommodate a narrative that incarnates the convergence schema (Gick and Holyoak, 1980, 1983), and that this analogical schema can then be used in problem solving for the general story and the fire-chief story, both of which are resolved based on the convergence schema. This demonstrates how language may structure events in the service of analogical problem solving, via the narrative construction. Perhaps the most interesting application of such mechanisms is the use of narrative as a means to structure social interaction and to learn patterns of behavior that lead to the construction of a folk psychology, according to the narrative practice hypothesis (Hutto, 2007; Gallagher and Hutto, 2008). Interestingly, developmental studies describe correlations between mothers' explanatory, causal and contrastive talk about mental states and theory of mind processing as assessed by a false belief task (Slaughter et al., 2007). We believe that our modeling framework is well suited to the exploration of this hypothesis, and this will occupy our future research.

## Author contributions

All authors made substantial contributions to the conception or design of the model, analysis, or interpretation of data for the work. All authors participated in drafting the work or revising it critically for important intellectual content. All authors have provided final approval of the version to be published. All authors agree to be accountable for all aspects of the work in ensuring that questions related to the accuracy or integrity of any part of the work are appropriately investigated and resolved.

### Conflict of interest statement

The authors declare that the research was conducted in the absence of any commercial or financial relationships that could be construed as a potential conflict of interest.

## Footnotes

^1^https://www.youtube.com/playlist?list=PL1e-_QtoQfPzYeLuds1wc0gdnfsbzgz_l
